# Impact of androgen deprivation therapy on mortality of prostate cancer patients with COVID-19: a propensity score-based analysis

**DOI:** 10.1186/s13027-021-00406-y

**Published:** 2021-11-25

**Authors:** Mateus Bringel Oliveira Duarte, Frederico Leal, Juliana Luz Passos Argenton, José Barreto Campello Carvalheira

**Affiliations:** 1 Division of Oncology, Department of Anesthesiology, Oncology and Radiology, School of Medical Sciences, State University of Campinas (UNICAMP), Campinas, SP Brazil; 2 Uberlândia Cancer Hospital, Federal University of Uberlândia, UFU, Uberlândia, MG Brazil; 3 Fundação de Desenvolvimento da Universidade Estadual de Campinas (FUNCAMP), Campinas, SP Brazil

**Keywords:** COVID-19, Androgen antagonists, Androgen receptor antagonists, Antineoplastic agents

## Abstract

**Background:**

Previous studies hypothesized that androgen deprivation therapy (ADT) may reduce severe acute respiratory syndrome coronavirus 2 (SARS-COV2) infectivity. However, it is unknown whether there is an association between ADT and a higher survival in prostate cancer patients with COVID-19.

**Methods:**

We performed a retrospective analysis of prostate cancer (PC) patients hospitalized to treat COVID-19 in Brazil’s public health system. We compared patients with the active use of ADT versus those with non-active ADT, past use. We constructed propensity score models of patients in active versus non-active use of ADT. All variables were used to derive propensity score estimation in both models. In the first model we performed a pair-matched propensity score model between those under active and non-active use of ADT. To the second model we initially performed a multivariate backward elimination process to select variables to a final inverse-weight adjusted with double robust estimation model.

**Results:**

We analyzed 199 PC patients with COVID-19 that received ADT. In total, 52.3% (95/199) of our patients were less than 75 years old, 78.4% (156/199) were on active ADT, and most were using a GnRH analog (80.1%; 125/156). Most of patients were in palliative treatment (89.9%; 179/199). Also, 63.3% of our cohort died from COVID-19. Forty-eight patients under active ADT were pair matched against 48 controls (non-active ADT). All patients (199) were analyzed in the double robust model. ADT active use were not protective factor in both inverse-weight based propensity score (OR 0.70, 95% CI 0.38–1.31, *P* = 0.263), and pair-matched propensity score (OR 0.67, 95% CI 0.27–1.63, *P* = 0.374) models. We noticed a significant imbalance in the propensity score of patients in active and those in non-active ADT, with important reductions in the differences after the adjustments.

**Conclusions:**

The active use of ADT was not associated with a reduced risk of death in patients with COVID-19.

**Supplementary Information:**

The online version contains supplementary material available at 10.1186/s13027-021-00406-y.

## Introduction

The pandemic caused by the new severe acute respiratory syndrome coronavirus 2 (SARS-CoV2) virus has been a challenge to health systems worldwide [1]. World Health Organization already recognized more than 5 million deaths [2]. To date, limited drugs have proved survival benefits in clinical trials for coronavirus disease 2019 (COVID-19) [3]. Recent trials demonstrated that COVID-19 vaccines offer great hope for the pandemic control [4–6], although the equitable distribution of vaccines and long term effects still`s a major concern [7]. Strategies prioritizing multiple approaches are critical in the war against the COVID-19 pandemic and recent evidence demonstrate that they are associated with better clinical outcomes [8].

From the early studies of COVID-19, there has been a clear susceptibility of the male sex to increased severity and mortality [9]. Recent reports have suggested that sex hormones play an important role in this finding [10]. For instance, conditions associated with hyperandrogenic states, such as androgenic alopecia, were associated with severe presentations of COVID-19 [11, 12]. Mechanistically, in vitro studies demonstrated that androgen blockade could reduce the expression of angiotensin-converting enzyme 2 (ACE2) and transmembrane serine protease 2 (TMPRSS2) receptors, resulting in reduced infectivity by the SARS-CoV2 virus [13, 14]. Currently, several trials are exploring androgen blockade as an alternative for the treatment and prevention of COVID-19 [15].

Prostate cancer (PC) represents a singular situation in COVID-19. TMPRSS2, a critical pathway to SARS-CoV2 infectivity, is also one of the most frequently mutated genes in PC. In theory, some investigators have pointed out that this could lead to an increased susceptibility to severe COVID-19 [16]. At the same time, PC patients are submitted to long periods of androgen deprivation therapy (ADT) [17], which is considered safe and its use is adequate during pandemic, furthermore it could lead to reduced infectivity and severity of COVID-19 [18–20]. This hypothesis was initially tested in Italian populations, where PC patients in ADT presented lower infection rates compared to other cancer populations [21], although other studies reported different results [22]. Herein, we investigated the influence of ADT in prostate cancer patients with COVID-19 on survival outcomes.

## Materials and methods

### Design

To investigate the influence of ADT in PC patients with COVID-19, we performed a retrospective cohort study linking COVID-19 databases with outpatient treatment databases from the Brazilian Unified Health System. In Brazil, all cases of suspected flu-like syndrome requiring hospitalization should be notified to SIVEP (Flu Epidemiologic Surveillance Information System) according to federal law. Since the start of the pandemic, the Brazilian Ministry of Health has adopted the SIVEP to account for COVID-19 information. We chose to analyze only cases with a reported positive SARS-COV2 PCR test. Intending to improve data quality, we selected only patients with COVID-19 reported by oncologic or academic hospitals. We also excluded patients with outpatient treatment, puerperal and pregnant women, patients less than 18 years old, with missing information on sex and age, and those without defined outcomes (in course hospitalization). Hospital discharge and death were defined as possible outcomes.

The variables age, presence of comorbidities (heart disease, asthma, chronic lung disease, nephropathy, and neurologic disease), Brazilian region of residence (Southeast vs. non-Southeast), and clinical outcome were retrieved from SIVEP. Cancer information was not mandatory information in SIVEP; thus, we found this information through an active search of comorbidities reported in free space in the SIVEP form. We excluded patients without a reported X-ray or radiologic information, assuming that all patients should have performed at least one radiological assessment during the hospitalization. Afterwards, we defined missing comorbidity information as an absence.

To obtain oncological information about the COVID-19 cases, we performed a linkage between SIVEP, SIA (Outpatient Information System), and SIH (Hospitalization Information System) databases. In Brazil, all outpatient treatments performed in the Unified Health System are registered in SIA. This system was initially created for reimbursement, but also contains information about clinical stage, primary site, line of treatment, and the scheme of treatment performed. SIH is similar to SIA, but includes information about in-hospital treatments, and also accounts for information in the diagnosis, procedures performed during hospitalization, outcomes, and some epidemiological information. As SIVEP is a primary dataset of hospitalized patients, those patients treated in Brazilian Unified Health System are in SIH as well. We performed the first linkage between SIVEP and SIH to improve the overall pool of variables to matching (adding zip postal code, and orchiectomies performed). The second linkage was performed between SIVEP/SIH and SIA. We used the second linkage to extract the oncological variables and ADT types. The linkages were deterministic and involved the variables birth date, sex, available dates (hospitalization, discharge, and admission to intensive care units), institution of code, city of residence, and zip code. After every phase of the linkage, the investigator assessed false matches confronting the available information (i.e.: primary site described in SIVEP against described in SIA). The SIA and SIH were limited to treatments performed between January 2018 and the last available dataset (ends of 2020). Finally, we select patients that have performed ADT to PC treatment. This methodology was previously described in another work by our group [23].

### Analysis

The SIVEP variables were considered positive if reported, otherwise, they were considered negative. Age was categorized as above or below 75 years, based on the overall median of the selected cohort. We defined those patients that used invasive mechanical ventilation as a critical presentation. Patients were also grouped according to the use or not of GnRH analogs, the line of treatment (palliative and non-palliative), and active or inactive use of ADT based on the past 2 months. Orchiectomy was considered an active treatment.

### Statistical methods

In the statistical analysis, we first compared the distribution of variables between survival and non-survival and then assessed them using an Exact Fisher test. Then, we performed a multivariate logistic regression model, with backward elimination, selecting variables with a minimum significance of 0.10.

To specifically test the effect of the active (versus non-active) use of ADT, we applied two propensity score models. In the first model, we used a pair matching process with a 1:1 ratio, with all variables used. We accessed this result with a conditional logistic regression between the paired groups.

In the second propensity score model, we performed a double robust estimation with the inverse weighting of the propensity score. In this model, we used all variables for the propensity score estimation, but only variables selected in the first logistic regression with backward elimination (performed before the first propensity score) were selected for outcome-based statistics. This model was assessed with a Wald test [24–26].

All values with a statistical significance superior to P < 0.05 were accepted. The work was performed in the software SAS Institute Inc., Cary, NC, USA. The project was submitted and approved by our institutional ethics (Comitê de Ética em Pesquisa (CEP) da Universidade Estadual de Campinas) commitment and the consent form was waived.

## Results

**Fig. 1:**
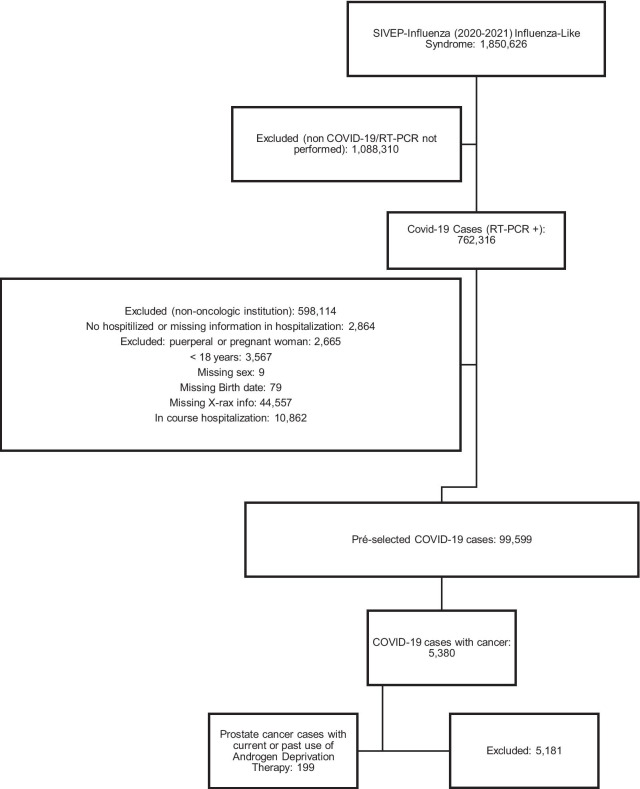
Data flow-chart

As of 26 April 2021, there were 1,850,626 cases of flu-like reported in SIVEP, and 762,316 with a positive RT-PCR test for SARS-COV2. After exclusions, we identified that 199 patients had performed ADT for prostate cancer treatment. Figure 1 summarizes the data flow-chart. As shown in Table 1, most of our patients were younger than 75 years old (52.3%; 104/199) and were from the Southeast region of Brazil (60.8%; 121/199). Heart disease was the most commonly reported comorbidity (40.7%; 81/199), followed by diabetes (24.6%; 49/199). 78.4% (156/199) were actively using ADT at the moment of the COVID-19 infection. LHRH analog was present in most of current ADT use (80.1%; 125/156). The majority were in the palliative line of treatment (89.9%; 179/199).

**Table 1:** Selected clinical characteristics according to hospital discharge or death and multivariate logistic regression analysis with and without inverse weight propensity score adjustment with death as outcome

Characteristic	Alive (n = 67)	Death (n = 132)	All patients (n = 199)	*P*^a^	OR UNI (95% CI)	*P*^b^	OR MV (95% CI)	P^b^	OR IWDRE (95% CI)	*P*^b^
*Age*										
≤ 75 years	40 (59.7)	64 (48.5)	104							
> 75 years	27 (40.3)	68 (51.5)	95	0.176	1.57 (0.87–2.86)	0.136	1.63 (0.89–2.99)	0.115	1.03 (0.56–1.91)	0.924
*Comorbidities*										
Heart disease	25 (37.3)	56 (42.4)	81	0.543	1.24 (0.68–2.26)	0.488				
Diabetes	16 (23.9)	33 (25.0)	49	1.000	1.06 (0.54–2.11)	0.863				
Neurologic disease	2 (3.0)	9 (6.8)	11	0.340	2.38 (0.5–11.33)	0.277				
Asthma	2 (3.0)	4 (3.0)	6	1.000	1.02 (0.18–5.69)	0.986				
Chronic lung disease	5 (7.5)	12 (9.1)	17	0.794	1.24 (0.42–3.68)	0.698				
Nephropathy	4 (6.0)	17 (12.9)	21	0.151	2.33 (0.75–7.22)	0.143	2.06 (0.65–6.57)	0.22	2.2 (0.65–7.36)	0.203
Critical presentation	3 (4.5)	60 (45.4)	63	< 0.001						
Southeast	45 (67.2)	76 (57.6)	121	0.220	0.66 (0.36–1.23)	0.192	0.64 (0.34–1.2)	0.166	0.3 (0.15–0.61)	0.001
Active ADT	56 (83.6)	100 (75.8)	156	0.274	0.61 (0.29–1.31)	0.208	0.63 (0.29–1.37)	0.241	0.7 (0.38–1.31)	0.263
Palliative treatment	61 (91.0)	118 (89.4)	179	0.807	0.83 (0.3–2.27)	0.715				

ADT, Androgen deprivation therapy; OR, odds ratio; UNI, Univariate; MV, Multivariate; IWDRE, Inverse weight double robust estimation

^a^Fisher exact test

^b^Wald test

Overall, 66.3% (132/199) of our cohort died. In our univariate logistic regression, no variable was associated with mortality. In the first multivariate logistic regression analysis, the active use of ADT (OR 0.63, CI 95% 0.29–1.37, *P* = 0.241) remained without association to survival.

**Fig. 2:**
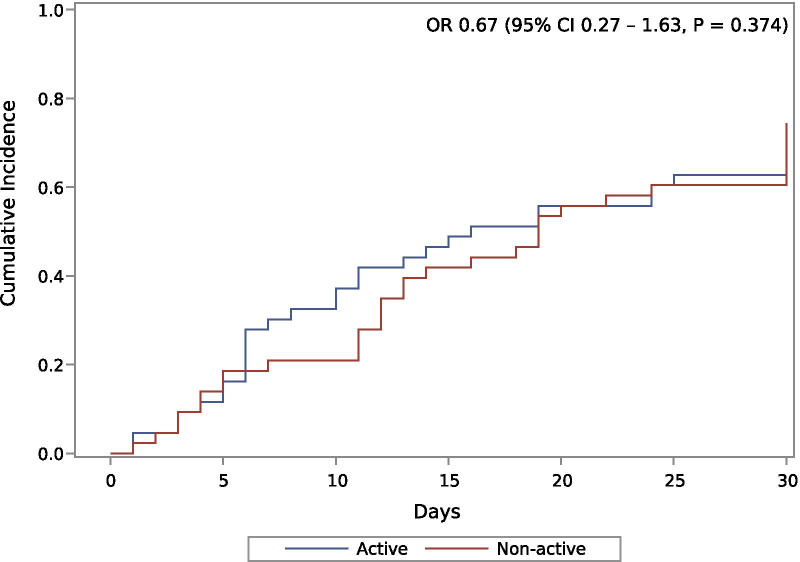
Pair-matched propensity score-based cumulative incidence of deaths. Statistics from this graph were derived from the pair-matched propensity score. Detailed in Additional file 1: Table S1. OR: Odds ratio

In the propensity score-based pair matching, we found that active use of ADT was not associated to mortality (OR 0.67, 95% CI 0.27–1.63, *P* = 0.374). Figure 2 shows the cumulative incidence of deaths of prostate cancer patients with COVID-19 according to the use of ADT after pair matching. Of note, in the propensity score analysis, we observed a significant imbalance between the variables before starting the adjustments (Additional file 1: Table S1, Additional file 2: Fig. S1, Additional file 3: Fig. S2). Even with a reduction of 84.5%, our matching algorithm presented difficulty to balance some variables (i.e., Critical Presentation).

**Fig. 3:**
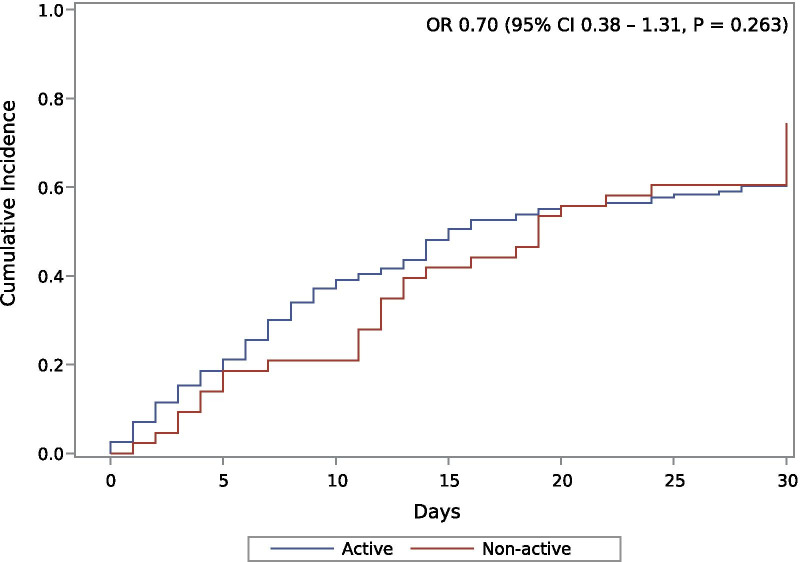
Cumulative incidence of deaths. Statistics from this graph were derived from the double robust estimation with inverse weight of the propensity scores. OR: Odds ratio

In our second propensity score model, using double robust estimation with inverse weight, we confirmed the previous findings that current ADT (OR 0.70 95% CI 0.38–1.31, *P* = 0.263) was not associated to survival outcomes, Fig. 3. After inverse weigh adjustment, we also found that southeast region was a protective factor (OR 0.30 95% CI 0.15–0.61, *P* = 0.001), Table 2. Additional file 4: Figure S3 presents the distribution of weights between the analyzed groups.

**Table 2:** Selected clinical characteristics according to active and non-active androgen deprivation therapy and propensity score based inverse weight double robust estimation with death as outcome

Characteristic	Active (n = 156)	Non-active (n = 43)	Total (n = 199)	*P*^a^	Standardized difference	
					Unweighted	Weighted
*Age*						
≤ 75 years	79 (50.6)	25 (58.1)	104 (52.3)	0.395	− 0.151	0.113
> 75 years	77 (49.4)	18 (41.9)	95 (47.7)			
*Comorbidities*						
Heart Disease	60 (38.5)	21 (48.8)	81 (40.7)	0.226	0.210	− 0.018
Diabetes	37 (23.7)	12 (27.9)	49 (24.6)	0.555	0.096	− 0.031
Neurologic disease	8 (5.1)	3 (7)	11 (5.5)	0.706	0.078	0.141
Asthma	3 (1.9)	3 (7)	6 (3)	0.116	0.247	− 0.006
Chronic lung disease	14 (9)	3 (7)	17 (8.5)	1	− 0.074	0.065
Nephropathy	12 (7.7)	9 (20.9)	21 (10.6)	0.022	0.385	− 0.023
Critical presentation	48 (30.8)	15 (34.9)	63 (31.7)	0.711	0.088	− 0.052
Southeast	94 (60.3)	27 (62.8)	121 (60.8)	0.861	0.052	0.063
Palliative treatment	142 (91)	37 (86.1)	179 (89.9)	0.39	− 0.157	0.011
*Outcome*						
Hospital discharge	56 (35.9)	11 (25.6)	67 (33.7)	0.274		
Death	100 (64.1)	32 (74.4)	132 (66.3)			

^a^Fisher exact test

## Discussion

Due to the long periods of ADT, PC patients represent a powerful model with which to study sex hormone suppression in patients with COVID-19. In our dataset, we found that the active use of ADT was not associated with lower COVID-19 mortality. The seminal work of Montopoli et al. [21] reported that PC patients using ADT had a 4 times lower risk of COVID-19 infection than others with PC. Although the work suggests a protective role of ADT in COVID-19 infection, the authors did not evaluate the impact of ADT in survival or disease severity. The present data did not support the protective effect of ADT to an inpatient scenario, since ADT use was not associated with favorable COVID-19 prognosis.

In vitro models demonstrated that there is an intrinsic relationship between androgen receptors and the expression of ACE2 and TMPRSS2 proteins, a critical pathway to COVID-19 infection [13, 14]. Both TMPRSS2 and ACE2 are indispensable to SARS-COV2 infection [27–29]. Interestingly, the TMPRSS2:ERG fusion gene is a common PC driver mutation in PC [30, 31], which represents a possible bridge between the two entities [19]. Indeed, Qiao et al. showed that androgen receptor blockade could not only reduce the expression of ACE2 and TMPRSS2 in a murine model but also reduce the in vitro infectivity rate of COVID-19 [13]. There are several clinical trials ongoing exploring ADT and TMPRSS2 blockers (Camostat and Nafamostat) in the treatment of COVID-19 [15]. In contrast to the preclinical findings Caffo et al. [32] did not observe reduced mortality associated with ADT in patients with metastatic castration-resistant PC (mCRPC). Accordingly, we did not observe that the line of treatment was not statistically associated with survival, although the subgroup of mCRPC was under-represented in our cohort.

Previous studies have demonstrated a strong association between sex hormones [33, 34], GnRH [35], and the immune system. Clinical models demonstrated that lymphocytes express the GnRH receptors and can interact with its feedbacks loops [36–38]. We cannot rule out the possibility that GnRH analogs could down-regulate hyper-inflammatory states of COVID-19. GnRH analogs already were used to treat other hyper-inflammatory conditions [39]. In our cohort, the absolute number of patients using a GnRH analog limited our ability of analyze this variable in a stable model. Thus, we do not discard the possibility of a protective effect of GnRH analogs against COVID-19.

Of note, oncological cohorts of PC did not present survival outcomes data of COVID-19 according to the use of ADT. Lee et al. [40]. did not report prostate cancer to be a protective factor against COVID-19, although the ADT per se was not evaluated in their analysis. On the other hand, the CCC19 group reported that the recent use of cancer hormone therapy was a protective factor [41], but the authors did not investigate the ADT itself. In our cohort, we noted a significant imbalance between the groups of prostate cancer patients in active ADT versus those in non-active ADT. Therefore, we recommend caution when evaluating ADT in observational studies. Even, using more robust propensity score-based models it was difficult to adjust all the imbalances between the groups. Importantly, the OR, independent of the statistical method used, remained not statistically significant. Consistent with our data, a recent metanalysis showed that patients receiving ADT did not have a reduced COVID-19 infection risk as well as COVID-19 hospitalization risk, ICU admission, and Mortality risk [42].

In general, the Brazilian SIVEP cohort presents a higher mortality [43, 44]. We believe that this finding represents the overall severity of Brazilian COVID-19 patient’s hospitalizations, as discussed elsewhere [23]. Allied to this, PC is a condition of elderly patients, with an established high risk of COVID-19 infection due to the high prevalence of comorbidities in this group of patients [45]. Although we believe that our statistical methods, using propensity score approaches, could improve our quality of analysis, important limitations of our work are the retrospective design and the inherent risk of bias in the data generation process. We also used a deterministic linkage process based on cancer treatment; thus, we do not discard the possibility of selection bias that prioritizes selection of those in active treatment.

## Conclusion

Our study indicates that PC patients in active ADT does not have lower mortality from COVID-19.

## Supplementary Information

**Additional file 1**. Supplemental Table 1. Baseline variables of active and non-active androgen deprivation therapy groups and standardized mean differences after propensity score-based pair matching.

**Additional file 2**. **Figure S1**. Standardized mean difference. The mean difference represents the difference between propensity score inside the variable before and after pair matching.

**Additional file 3**. **Figure S2**. Cumulative distribution of logit propensity score. The graphs summarize the cumulative distribution of logit propensity score, as well as the difference between active and non-active groups before and after matching.

**Additional file 4**. **Figure S3**. Density of propensity score distribution. The figure summarizes the distribution of propensity score applied in the double robust estimation model according to the use of androgen deprivation therapy (ADT).

## Data Availability

The datasets during and/or analyzed during the current study available from the corresponding author on reasonable request.
