# Supplementary material for: Impact of androgen deprivation therapy on mortality of prostate cancer patients with COVID-19: a propensity score-based analysis
Source: Infect Agent Cancer. 2021 Nov 25;16:66. doi: 10.1186/s13027-021-00406-y (PMC8614632; doi:10.1186/s13027-021-00406-y)
Supplement: Supplementary file 1 — Additional file 1. Supplemental Table 1. Baseline variables of active and non-active androgen deprivation therapy groups and standardized mean differences after propensity score-based pair matching. [file 13027_2021_406_MOESM1_ESM.docx]

| Characteristic | Active (n = 48) | Non-active (n = 48) | P^*^ | Standardized Mean Differences | |
| --- | --- | --- | --- | --- | --- |
| Age |  |  |  | **Unmatched** | **Matched** |
| ≤ 75 years | 21 (48.8) | 25 (58.1) | 0.517 | 0.151 | 0.187 |
| > 75 years | 22 (51.2) | 18 (41.9) |  |  |  |
| Comorbidities |  |  |  |  |  |
| Heart Disease | 22 (51.2) | 21 (48.8) | 1.000 | -0.21 | 0.047 |
| Diabetes | 12 (27.9) | 12 (27.9) | 1.000 | -0.096 | 0 |
| Neurologic disease | 3 (7) | 3 (7) | 1.000 | -0.078 | 0 |
| Asthma | 2 (4.7) | 3 (7) | 1.000 | -0.247 | -0.114 |
| Chronic lung disease | 4 (9.3) | 3 (7) | 1.000 | 0.074 | 0.086 |
| Nephropathy | 11 (25.6) | 9 (20.9) | 0.799 | -0.385 | 0.135 |
| Critical Presentation | 11 (25.6) | 15 (34.9) | 0.482 | -0.088 | -0.198 |
| Southeast | 30 (69.8) | 27 (62.8) | 0.649 | -0.052 | 0.143 |
| Palliative Treatment | 39 (90.7) | 37 (86.1) | 0.738 | 0.157 | 0.146 |
| Outcome |  |  |  |  |  |
| Hospital Discharge | 15 (34.9) | 11 (25.6) | 0.482 |  |  |
| Death | 28 (65.1) | 32 (74.4) |  |  |  |
| Logit Propensity Score |  |  |  | 0.562 | 0.087 |

**Supplemental Table 1.** Baseline variables of active and non-active androgen deprivation therapy groups and standardized mean differences after propensity score-based pair matching.

Legends: ^*^Fisher’s exact test.
