# Supplementary figures and images for: Impact of androgen deprivation therapy on mortality of prostate cancer patients with COVID-19: a propensity score-based analysis
Source: Infect Agent Cancer. 2021 Nov 25;16:66. doi: 10.1186/s13027-021-00406-y (PMC8614632; doi:10.1186/s13027-021-00406-y)

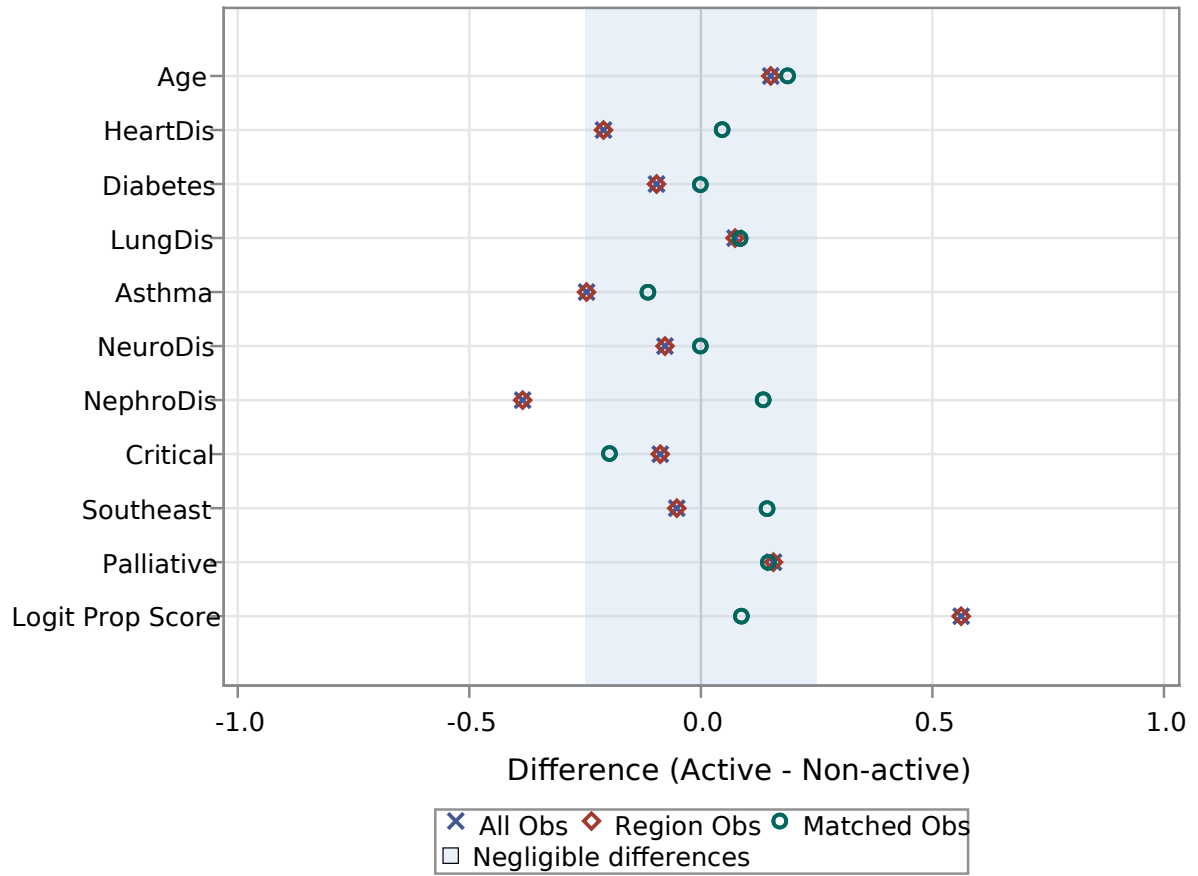

Supplement: Supplementary file 2 — Additional file 2. Figure S1. Standardized mean difference. The mean difference represents the difference between propensity score inside the variable before and after pair matching. [file 13027_2021_406_MOESM2_ESM.pdf]

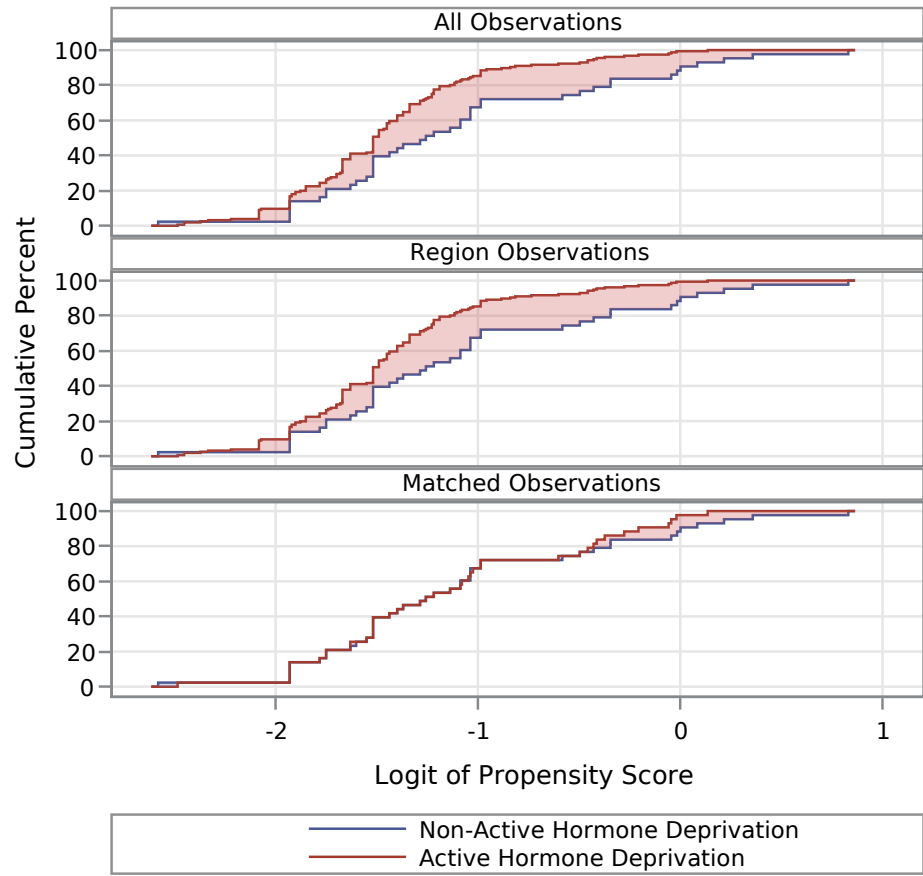

Supplement: Supplementary file 3 — Additional file 3. Figure S2. Cumulative distribution of logit propensity score. The graphs summarize the cumulative distribution of logit propensity score, as well as the difference between active and non-active groups before and after matching. [file 13027_2021_406_MOESM3_ESM.pdf]

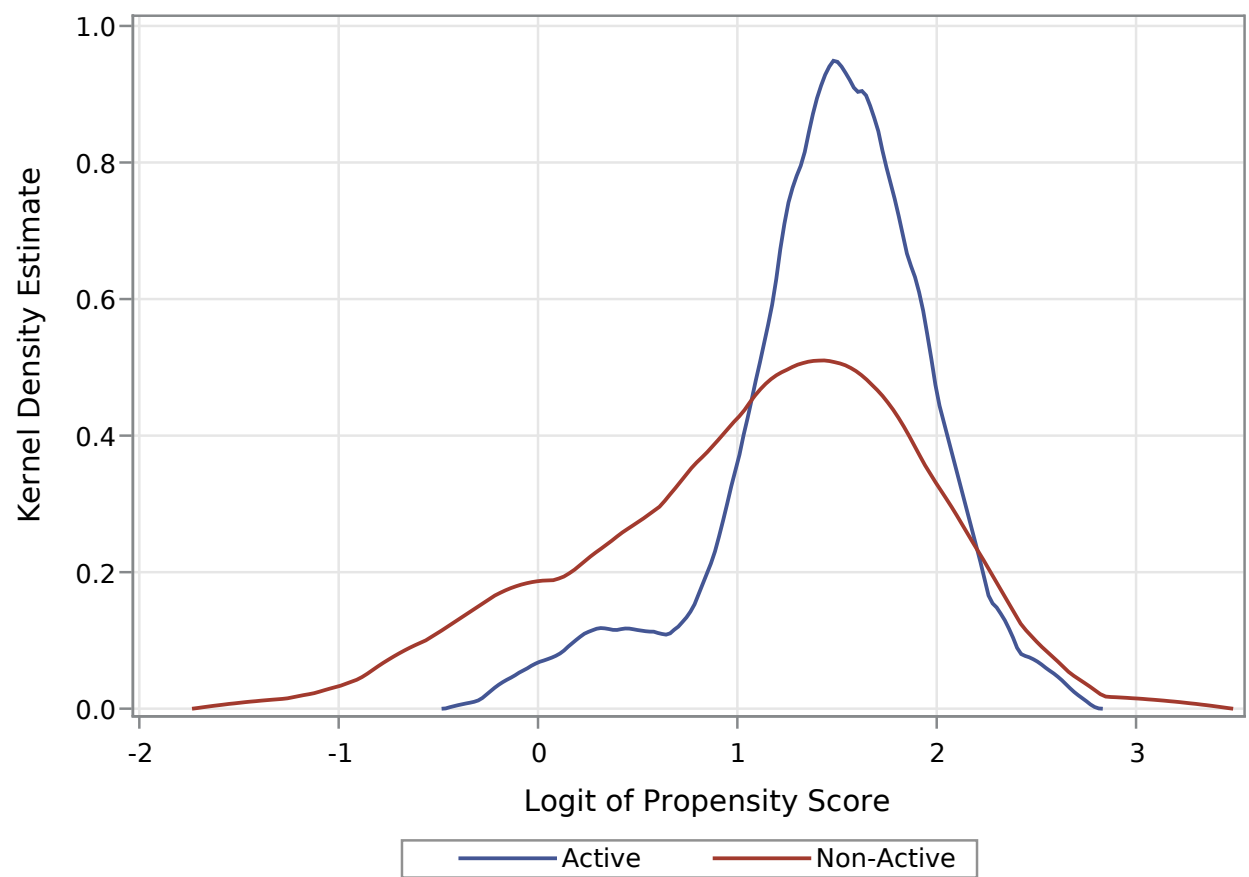

Supplement: Supplementary file 4 — Additional file 4. Figure S3. Density of propensity score distribution. The figure summarizes the distribution of propensity score applied in the double robust estimation model according to the use of androgen deprivation therapy (ADT). [file 13027_2021_406_MOESM4_ESM.pdf]
